# Thuringiensin: A Thermostable Secondary Metabolite from *Bacillus thuringiensis* with Insecticidal Activity against a Wide Range of Insects

**DOI:** 10.3390/toxins6082229

**Published:** 2014-07-25

**Authors:** Xiaoyan Liu, Lifang Ruan, Donghai Peng, Lin Li, Ming Sun, Ziniu Yu

**Affiliations:** 1 Hubei Biopesticide Engineering Research Center, Hubei Academy of Agricultural Sciences, Wuhan 430064, China; E-Mail: xiaoyanliu6613@163.com; 2 State Key Laboratory of Agricultural Microbiology, College of Life Science and Technology, Huazhong Agricultural University, Wuhan 430070, China; E-Mails: ruanlifang@mail.hzau.edu.cn (L.R.); gongeleven2005@webmail.hzau.edu.cn (D.P.); lilin@mail.hzau.edu.cn (L.L.)

**Keywords:** thuringiensin, *Bacillus thuringiensis*, insecticidal mechanism, genetic determinants

## Abstract

Thuringiensin (Thu), also known as β-exotoxin, is a thermostable secondary metabolite secreted by *Bacillus thuringiensis*. It has insecticidal activity against a wide range of insects, including species belonging to the orders Diptera, Coleoptera, Lepidoptera, Hymenoptera, Orthoptera, and Isoptera, and several nematode species. The chemical formula of Thu is C_22_H_32_O_19_N_5_P, and it is composed of adenosine, glucose, phosphoric acid, and gluconic diacid. In contrast to the more frequently studied insecticidal crystal protein, Thu is not a protein but a small molecule oligosaccharide. In this review, a detailed and updated description of the characteristics, structure, insecticidal mechanism, separation and purification technology, and genetic determinants of Thu is provided.

## 1. Introduction

*Bacillus thuringiensis* is a sporogenic soil bacterium that produces insecticidal crystal (Cry) proteins that are highly specific for the larvae of various insect pests [1]. Because of this capability, *B. thuringiensis* has been widely used in biological pest control [2,3]. In addition to Cry proteins, several other insecticidal factors in *B. thuringiensis* have been observed, such as thuringiensin (Thu), vegetative insecticidal proteins (VIP) [4], secret insecticidal protein (Sip) [5], zwittermicin A (ZwA) [6], Mtx-like toxin [7], and Bin-like toxin [8].

Thu, also known as β-exotoxin, is a thermostable exotoxin produced by *B. thuringiensis*, a broad-spectrum vegetative factor expressed during vegetative growth and secreted in the supernatant [9]. Thu was first identified by McConnell and Richards in 1959 following injection of autoclaved supernatant from liquid *B. thuringiensis* culture into several species of insect, which resulted in the death of the insects [10]. This discovery stimulated the study of Thu in several laboratories.

To date, researchers have mainly focused on purification, detection, fermentation, insecticidal activity, and toxicity of Thu, while genetic determinants have remained under-studied. In the present review, we discuss current developments in the study of the characteristics, structure, insecticidal spectrum and mechanism, security appraisal, separation and purification, and genetic determinants of Thu.

## 2. Discussion

### 2.1. Characteristics and Structure of Thu

**Figure 1:**
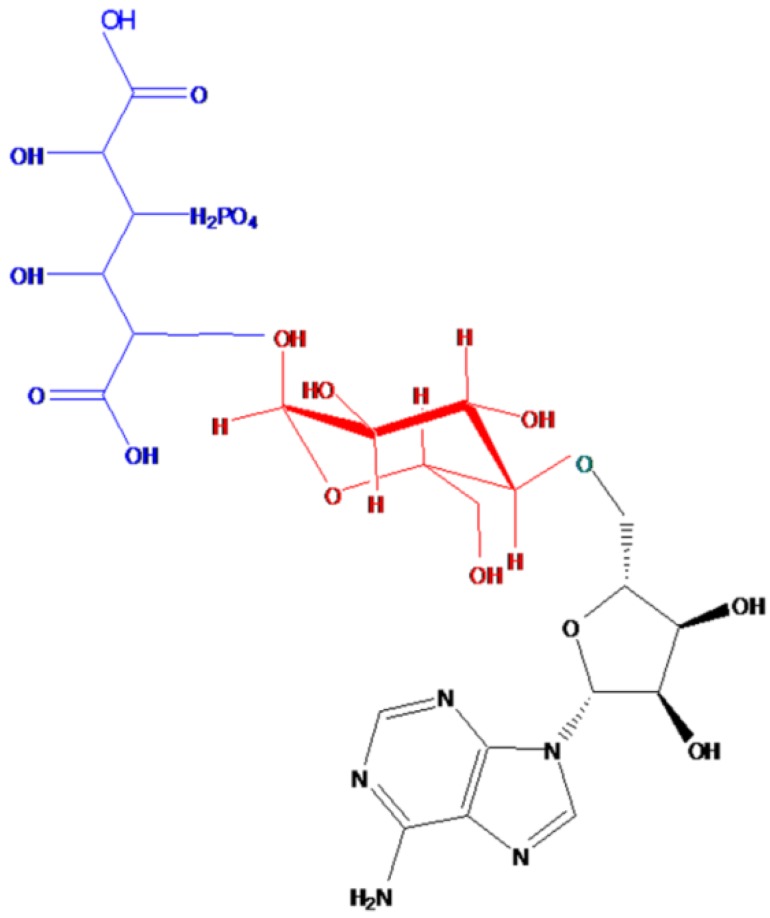
Structure of thuringiensin [11].

Unlike the insecticidal Cry protein, Thu is a nonspecific small molecule oligosaccharide composed of adenosine, glucose, phosphoric acid, and gluconic diacid at a molecular ratio of 1:1:1:1. The chemical formula is C_22_H_32_O_19_N_5_P, and the molecular weight is 701 Da. Thu is a thermostable exotoxin and retains its bioactivity at 121 °C for 15 min [11,12]. The chemical structure of Thu is shown in Figure 1.

### 2.2. Insecticidal Spectrum of Thu

Thu is toxic to species of the Diptera, Coleoptera, Lepidoptera, Hymenoptera, Orthoptera, and Isoptera insect orders, and several nematode species [13,14,15]. Toledo *et al.* (1999) found that Thu is highly toxic to three species of fruit fly, *Anastrepha ludens* (Loew), *Anastrepha obliqua* (Macquart), and *Anastrepha serpentina* (Wiedemann), with LC_50_ values of 0.641, 0.512, and 0.408 mg/cm^2^, respectively [13]. Zhang *et al.* (2000) examined the toxicity of Thu by feeding the purified Thu to *Helicoverpa armigera*, and found that Thu significantly restricts the growth of *H. armigera* [16]. In 2002, Tsuchiya *et al.* isolated 2652 *B. thuringiensis* strains from Japan, which were assessed for their toxicity towards the cigarette beetle *Lasioderma serricorne* (Coleoptera: Anobiidae). Results showed that Thu is responsible for the toxicity against the cigarette beetle [15]. Thu is also effective against plant parasitic nematodes [12].

### 2.3. Security Appraisal of Thu

The toxicity of Thu in mammals has been the subject of debate for some time. Studies to date have not clearly established whether Thu is toxic to humans, however it has been banned from public use based on World Health Organization advice [17]. In 1977, rats feeding on Thu showed no significant differences in chromosome aberration rates compared with the controls following analysis of bone marrow mid cells, myeloma cells, and blood cells [18]. In addition, Barjac *et al.* (1968) recycled mouse dung after the mice ingested food containing ^32^P-labelled Thu for 24 h. Thu was not observed in any organ, including the liver, heart, kidney, spleen, and adrenal gland [19].

However, several experiments have shown that Thu is toxic to mammals. In one experiment, rats were intratracheally inoculated with 0, 0.4, 0.8, 1.6, 3.2, 6.4, or 9.6 mg/kg body weight of Thu. The results indicated that the acute pulmonary LD_50_ of Thu in rats was 4.4 mg/kg. The treated rats presented abnormal histology, including disseminated inflammation in the bronchioles and alveoli, bronchial cellular necrosis on days 1 and 2, and areas of septal thickening with cellular infiltration and collagen deposits in the intestinal and alveolar spaces on days 4–5 [20]. Tsai *et al.* (2004) demonstrated that Thu could activate basal adenylate cyclase activity and increase cAMP concentrations in rat cerebral cortex or in a commercial adenylate cyclase [21]. Thu is generally regarded as a weak activator of adenylate cyclase or an inhibitor of forskolin-stimulated adenylate cyclase; therefore, changes in pulmonary oxidative-antioxidative status might have an important role in Thu-induced lung injury (2006) [22].

### 2.4. Insecticide Mechanism

The insecticidal mechanism of Thu is not fully understood. However, it is known to be an ATP analog [11] that interferes with RNA polymerase [23,24]. Thu inhibits the synthesis of RNA by competing with ATP on binding sites, affecting insect molting and pupation, and causing teratological effects at sublethal doses [25,26,27]. The disease symptoms caused by Thu in insects are different from those caused by insecticidal cry protein, and are only observed during insect molting and pupation. At the minimum effective dose of Thu, larvae develop and pupate normally, with only small pupae failing to undergo eclosion and only a few adults being teratological. In contrast, at higher doses of Thu, larval pupation is abnormal, or the larvae become unresponsive during molting and eventually die [28].

### 2.5. Separation and Purification of Thu

Several methods can be used to separate and purify Thu. Barjac *et al.* first purified Thu in 1966 using centrifugation method, and since then, many other purification methods have been established [29]. Kim *et al.* (1970) improved the initial technique by adding a CaCl_2_ precipitation step [30]. Membrane ultrafiltration technology has also been used to purify Thu. However, ultrafiltration has several disadvantages, including high cost, low efficiency, and extended duration. Tsun *et al.* (1999) used a micellar-enhanced ultrafiltration method to recover Thu. This method was significantly improved by the use of a spiral-wound membrane, which could be operated at a low transmembrane pressure drop. The surfactant cetylpyridinium chloride (CPC) was also added to the fermentation broth, which was passed through the ultrafiltration membrane and the retentate was collected. Maximum recovery of up to 99.3% was achieved [31]. However, this method has several disadvantages, such as restricted adsorption, toxicity of CPC, and micellar aggradation. Tzeng *et al.* (2001) developed a two-step method for recovery of Thu, including adsorbing it from the fermentation broth by calcium silicate and dissociating Thu by dibasic sodium phosphate. The obtained Thu could be further purified through semi-preparative high-performance liquid chromatography (HPLC) and electrodialysis to remove the excess salts from the recovered Thu solution [32]. To rapidly test Thu production from a large group of strains, Gohar *et al.* (2001) proposed a new HPLC procedure for quantification of this exotoxin in culture supernatants using acetone and acetonitrile. This method could detect levels as low as 0.3 μg/mL of Thu [33]. In addition, other methods have been developed, including bioassay, ion-exchange chromatography, spectrophotometry, reverse phase liquid chromatography, micellar electrokinetic capillary chromatography, and enzyme-linked immunosorbent assay [34,35,36,37]. De Rijk *et al.* (2013) established a confirmatory quantitative method for the determination of Thu in plant protection products and selected greenhouse crops based on liquid chromatography tandem mass spectrometry; the limit of quantitation was 0.028 mg/kg [38].

### 2.6. Genetic Determinants of Thu

Multiple studies on the regulatory mechanisms and genetic determinants involved in Thu production have been performed, and the main findings are discussed below.

#### 2.6.1. Relationship between Plasmids and Thu Production

Levinson *et al.* (1990) performed plasmid elimination in *B. thuringiensis* strain HD-2, followed by a transconjugation assay. The results showed that derivatives of HD-2 cured of a 75 MDa plasmid no longer produced Thu, whereas transconjugants that acquired the plasmid produced the toxin. Bioassays showed that only strains containing the 75-MDa plasmid were toxic to flies [34]. Perani *et al.* (1998) isolated *B. thuringiensis* from soil and found that up to 35% of the isolates possessed *cry1B*, and 58% of these produced Thu [39]. Espinasse *et al.* (2002) examined 640 natural isolates of *B. thuringiensis* and found that secretion of significant amounts of Thu was strongly associated with *cry1B* and *vip2*. They concluded that strains carrying *cry1B* and *vip2* also possess genetic determinants necessary to promote high levels of Thu production on the same plasmid [27]. Espinasse *et al.* (2002) reported that the mutant strain *B. thuringiensis* 407-1(Cry^−^)(Pig^+^), with no crystal gene, produced considerable amounts of Thu with a soluble brown melanin pigment. Using a mini-Tn10 transposon, they constructed a library of strain 407-1(Cry^−^)(Pig^+^) mutants, and screened for nonpigmented mutants with impaired Thu production. The results identified a genetic locus harboring two genes (*berA* and *berB*) that were essential for Thu production. BerA protein displayed significant similarity to the ATP-binding domains of the drug resistance and immunity (DRI) family of ATP-binding cassette proteins involved in the DRI family of bacteriocins and lantibiotics. BerB has six putative transmembrane helices, which probably constitute the integral membrane component of the transporter. This finding suggests that *berAB* confers Thu immunity in *B. thuringiensis* through active efflux of the molecule [40]. In 2004, the same group described the characterization and transcriptional analysis of a gene cluster, *sigW-ecfX-ecfY*, which was essential for Thu production in *B. thuringiensis* subsp. *thuringiensis* strain 407-1. Disruption of the *sigW* cluster resulted in nontoxic culture supernatants. The authors suggested that, in the wild-type 407 (Cry^+^) strain, Thu was produced from determinants located on a cry gene-bearing plasmid, and that *sigW* induced Thu production in *B. thuringiensis* in the absence of cry gene-bearing plasmids [41].

#### 2.6.2. The Synthesis Genes

**Figure 2:**
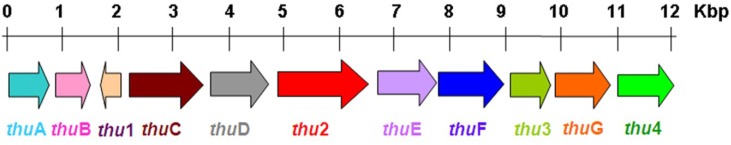
*thu* cluster. *thuA*: glucose-6-phosphate 1-dehydrogenase gene; *thuB*: racemase gene; *thuC*: PEP-protein phosphotransferase gene; *thuD*: UDP-glucose-6-dehydrogenase gene; *thuE*: shikimate kinase gene; *thuF*: glycosyltransferase gene; *thuG*: *N*-acyl-*D*-glucosamine 2-epimerase gene; *thu*1: putative exopolysaccharide polymerization protein gene; *thu*2: non-ribosomal peptide synthetase gene; *thu*3: bacterial gene of unknown function; *thu*4: SAM-dependent methyltransferase.

In 2010, our group used heterologous expression-guided screening in an *Escherichia coli*-*B. thuringiensis* shuttle bacterial artificial chromosome library to clone the intact Thu synthesis cluster. The Thu cluster was located on a 110-kb endogenous plasmid bearing the insecticide cry protein gene *cry1Ba* in *B. thuringiensis* strain CT-43. The cluster comprised 11 open reading frames and was further verified by genome sequencing data [42] (Figure 2).

Based on gene function, we deduced that *thu*s, *thu*C, and *thu*D possibly encode proteins responsible for the synthesis of the key precursor gluconic diacid (precursor A) from glucose 6-phosphate, while *thu*F and *thu*1 might encode proteins responsible for the assembly of Thu. The predicted Thu2 protein, which comprises an adenylation (A) domain, a condensation (C) domain, and an acyl carrier protein (ACP) domain, might serve as the location for the assembly of Thu. The *thu*E gene was predicted to encode the enzyme used in the final modification (phosphorylation), and *thu*3 likely encodes a protein responsible for releasing mature Thu. *In* *vivo* and *in vitro* activity assays showed that *thuE* is responsible for the phosphorylation of Thu at the final step [12].

#### 2.6.3. The Biosynthesis Pathway

**Figure 3:**
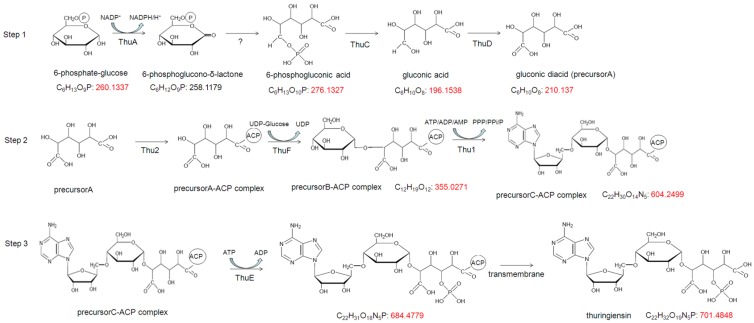
Schematic of the thuringiensin biosynthesis pathways. Step 1: synthesis of the key precursor, gluconic diacid (precursor A), by *thuA*, *thuC*, and *thuD*; Step 2: assembly of gluconic diacid, glucose, and adenosine by *thuF* and *thu1*; Step 3: phosphorylation of precursor C by ThuE. Numbers have been added to designate the molecular weight of each intermediate.

The Thu biosynthesis pathway was deduced according to the predicted gene functions (Figure 3).

Based on the structural formula, Thu is derived from four precursors: adenosine, glucose, one phosphate group, and gluconic diacid. The pathway could be divided into three steps. First is the synthesis of the key precursor, gluconic diacid (precursor A), using the products of *thu*s, *thu*C, and *thu*D. The initial substrate, glucose 6-phosphate, is oxidized to 6-phosphoglucono-δ-lactone by glucose-6-phosphate 1-dehydrogenase (ThuA), and subsequently hydrolyzed to 6-phosphogluconic acid. The dephosphorylation of 6-phosphogluconic acid is catalyzed by a PEP-protein phosphotransferase (ThuC). The resulting gluconic acid is subsequently oxidized to gluconic diacid by UDP-glucose dehydrogenase (ThuD), which can directly oxidize a hydroxyl group to a carboxyl (ic) group. In the second step, the assembly of gluconic diacid, glucose, and adenosine is performed by ThuF and Thu1. First, gluconic diacid is bound to the ACP region of the nonribosomal peptide synthetase Thu2. A hydroxyl group on the C-1 residue of gluconic diacid forms a thioester bond with a sulfhydryl group of the ACP protein. Next, a UDP-glucose moiety is added onto the gluconic diacid-ACP complex by glycosyltransferase ThuF, and a 1,5-glycosidic bond is formed between glucose and gluconic diacid. The resulting product (precursor B) is polymerized with the ribose of adenosine from ATP/ADP/AMP by an exopolysaccharide polymerization protein (Thu1). A 4,5-glycosidic bond is then formed between ribose and glucose, producing precursor C. Finally, precursor C is released from ACP. In the third and final step, precursor C is phosphorylated by ThuE, and mature Thu is released. The mature Thu molecule can be secreted by the cell. High performance liquid chromatography and ionization and time of flight mass spectrometry (LCMS-IT-TOF) was conducted to clarify the deduced pathway of Thu [12].

#### 2.6.4. Secretion and Immune Mechanism of Thu

A type IV-like secretion system (T4SS) was found on the plasmid harboring the *thu* cluster. Preliminary data showed that T4SSs and Thu3 were possibly involved in the secretion of Thu. The *thuE* gene in the *thu* cluster was proven to be responsible for Thu phosphorylation. This phenomenon occurred during the last step of the membrane translocation process. Mature Thu is excluded during the combination of Thu3 and T4SS. This mechanism protects the cell from possible damage by Thu [12].

## 3. Conclusions

Thu has long been considered an adenine nucleotide analog that interferes with RNA polymerase. It may possess some toxicity towards mammalian cells [6,7,11], and has therefore been banned from public use [17]. However, the most recent data shows that Thu is an adenine nucleoside oligosaccharide rather than an adenine nucleotide analog [12], as formerly believed. These new findings regarding the structure and characteristics of Thu may inspire further research on toxicity mechanisms and biosafety problems of Thu regarding mammalian cells.
